# An epigenetic toolbox for conservation biologists

**DOI:** 10.1111/eva.13699

**Published:** 2024-06-03

**Authors:** Alice Balard, Miguel Baltazar‐Soares, Christophe Eizaguirre, Melanie J. Heckwolf

**Affiliations:** ^1^ School of Biological and Behavioural Sciences Queen Mary University of London London UK; ^2^ Department of Biology University of Turku Turku Finland; ^3^ Department of Ecology Leibniz Centre for Tropical Marine Research Bremen Germany

**Keywords:** conservation biology, conservation genetics, ecological genetics, phenotypic plasticity, wildlife management

## Abstract

Ongoing climatic shifts and increasing anthropogenic pressures demand an efficient delineation of conservation units and accurate predictions of populations' resilience and adaptive potential. Molecular tools involving DNA sequencing are nowadays routinely used for these purposes. Yet, most of the existing tools focusing on sequence‐level information have shortcomings in detecting signals of short‐term ecological relevance. Epigenetic modifications carry valuable information to better link individuals, populations, and species to their environment. Here, we discuss a series of epigenetic monitoring tools that can be directly applied to various conservation contexts, complementing already existing molecular monitoring frameworks. Focusing on DNA sequence‐based methods (e.g. DNA methylation, for which the applications are readily available), we demonstrate how (a) the identification of epi‐biomarkers associated with age or infection can facilitate the determination of an individual's health status in wild populations; (b) whole epigenome analyses can identify signatures of selection linked to environmental conditions and facilitate estimating the adaptive potential of populations; and (c) epi‐eDNA (epigenetic environmental DNA), an epigenetic‐based conservation tool, presents a non‐invasive sampling method to monitor biological information beyond the mere presence of individuals. Overall, our framework refines conservation strategies, ensuring a comprehensive understanding of species' adaptive potential and persistence on ecologically relevant timescales.

## 1 INTRODUCTION

Climate change poses significant challenges, with many species and populations facing extinction (Díaz et al., 2019; Román‐Palacios & Wiens, 2020; Rosenberg et al., 2019; Scheele et al., 2019; Urban, 2015). While it is imperative to reduce and remove the threats biodiversity and ecosystems are facing, the rapid pace of environmental change calls for additional strategies that buffer detrimental effects and promote resilience. To be efficient conservation strategies of biodiversity must (i) delineate conservation units, (ii) determine individual health status, particularly in endangered and small populations, and (iii) estimate the **adaptive potential** of populations and communities (see Glossary in Table 1 for terms in bold). The adaptive potential is often defined as the ability of species/populations to respond to selection through phenotypic or molecular changes (Eizaguirre & Baltazar‐Soares, 2014). It also includes population structure, as migration maintains genetic diversity, and barriers to gene flow from habitat fragmentation accelerate species extinction (Crooks et al., 2017; Haddad et al., 2015). Conservation challenges call for the development of methods that assess overall biodiversity across all levels of biological organisation, from individuals, populations, species, and communities to ecosystems on ecologically relevant timescales. While gaining momentum (Formenti et al., 2022; Theissinger et al., 2023), genomic diversity used to be overlooked in biodiversity assessments and conservation initiatives (Stange et al., 2021). This oversight stems from the challenge of understanding where genomics can serve the conservation of endangered species, especially in situations where direct exploitations are at play and where threats manifest gradually, such as the perceived risks of climate change. There are currently many cases where genomic tools are part of conservation solutions, such as taxonomic identification, definition of population structure, population adaptive potential and biodiversity monitoring at large scales (Harrisson et al., 2014; Theissinger et al., 2023; Whiteley et al., 2015).

**TABLE 1:** Glossary for terms used in this review.

Term	Definition
5mC	Methylated form of the nucleic acid cytosine (C) on both DNA strands
Adaptive potential	The ability of species/populations to respond to selection by means of phenotypic or molecular changes (Eizaguirre & Baltazar‐Soares, 2014)
Biomarker	Measurement obtained directly from the organism, indicative of its physiological status. In an epigenetic context, this is the epigenetic state at one or few genomic loci
CpG sites	Genome regions where linear DNA (in the 5′ to 3′ direction) consists of a cytosine + guanine. Long strings of CG repeats are called CpG islands. Methylation is usually symmetrical (also occurring in the 3′ to 5′ strand)
Differentially methylated regions (DMRs)	Genomic regions where methylation levels differ at least between two organisms
DNA methylation	Biochemical mark that generally results from the process of adding a methyl group to the 5C position of the cytosine aromatic ring (5‐methylcytosine, 5mC). In the process of methylation and demethylation, intermediate steps are created that also harbor valuable information, e.g. 5‐hydroxymethylcytosine (5hmC). A methyl group can also occur on other nucleotides or in different methylation motifs (CG, CHH; CHG, etc.)
eDNA	Short expression for “environmental DNA”. Environmental DNA can be broadly defined as DNA that can be found outside biological entities and thus free in the environment
Epiallele	Alternative version of an epigenetic mark, which can be defined by (i) absence/presence of DNA methylation mark and/or (ii) differences in methylation ratio
epi‐eDNA	Epigenetic counterpart of the conventionally screened genomic DNA in environmental DNA studies
Epigenetic clock	Based on the seminal work of Horvath (2013), it refers to a multi‐tissue molecular tool that estimates the age of an organism by measuring the accumulation rate of epigenetic marks
Epigenetics	Regulatory changes in gene expression that are meiotically and/or mitotically heritable but occur without alterations to the DNA sequence itself. They typically are driven by histone modifications, DNA methylation and non‐coding RNA
Epigenome	Whole genome distribution of epigenetic marks
Epimutation	Change in methylation level of a given DNA sequence
Gene regulation	Process involving expression of a gene. It specifically relates to factors and mechanisms controlling timing, location (organ, tissue or cell) and gene product being produced
Human assisted evolution	The improvement of populations by enhancing their stress tolerance – also through genetic modifications
Maladaptation (maladaptive phenotypes)	Reverse of adaptation in the sense that evolved trait confers disadvantage in a given environment. Here “trait” may refer to alleles (that code for phenotype) or to the phenotype itself
Methylome	Whole genome distribution of cytosine methylated sites (5mC)
ncRNA	Non‐coding RNA. Broad term for RNAs that do not encode known proteins
Promoter region	DNA region located upstream of gene that mediates its expression

Given the pace at which threats progress, especially human‐mediated, it is likely that classic genomic tools may not detect early warning signals of at‐risk or declining populations. However, considering that nearly one‐third of all species are threatened with extinction (IUCN, 2022), time is of the essence. In this context, screening for **epigenetic** modifications − including DNA methylation, histone modifications, and non‐coding RNA expression − emerges as a timely complement to ongoing monitoring strategies. This is because epigenetic modifications can drive phenotypic plasticity, allowing adaptive phenotypes to arise more quickly to cope with changing conditions (e.g. Anastasiadi et al., 2021; Chang et al., 2020). Consequently, developing epigenetic tools will empower conservation biologists to monitor rapid, short‐term shifts in key traits of target populations, and communities, as they capture the links between individuals and their environment as well as developmental changes (Aguilera et al., 2010; Cavalli & Heard, 2019; Martin & Fry, 2018).

**FIGURE 1:**
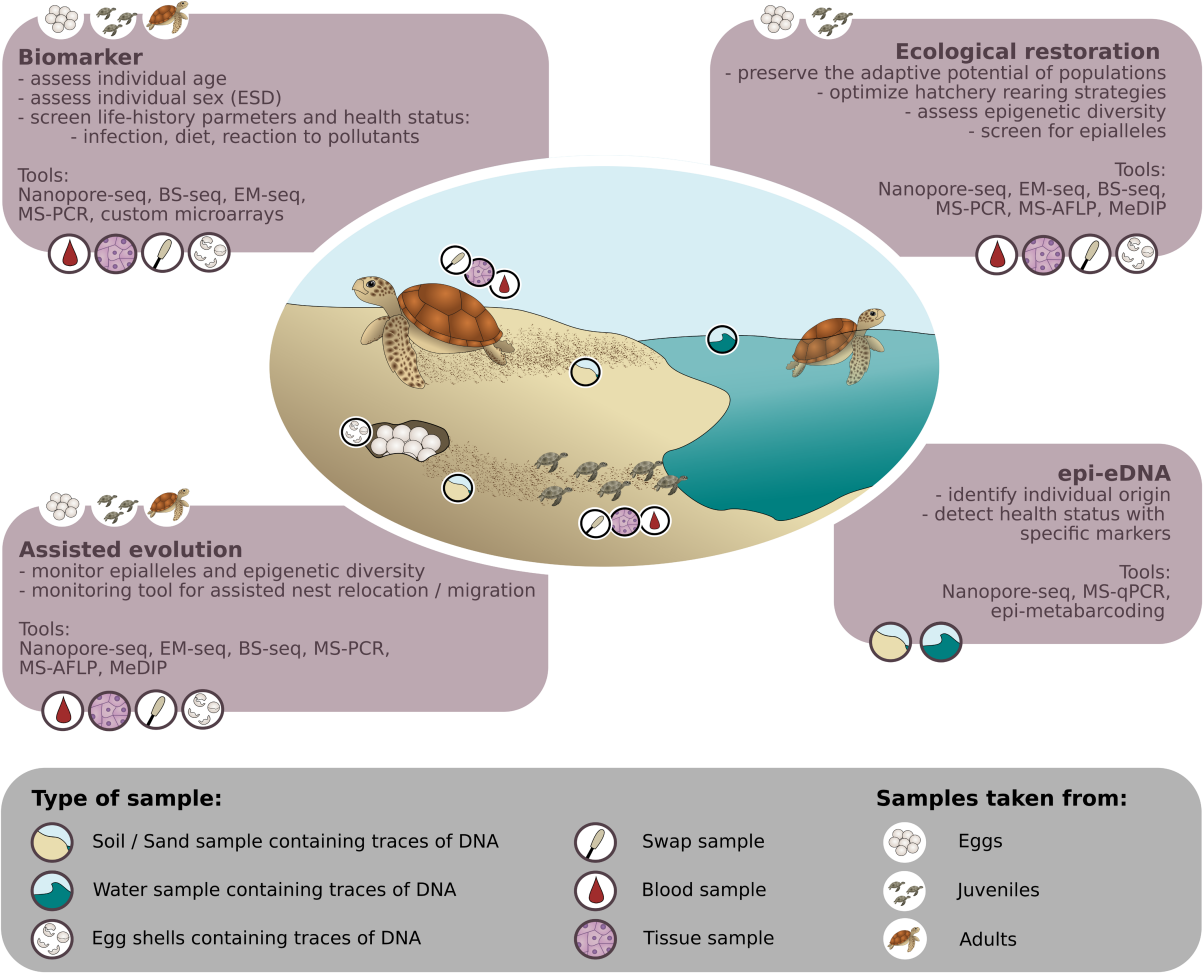
Example of DNA‐based epigenetic methods applied to the conservation of an endangered species. Here the focus is set on a species with temperature‐dependent sex determination.

While there are two groups of epigenetic tools, at the time of writing this review, one is readily available to conservation genomics. The one group in need of further development for applied conservation mostly deals with access to specific genomic regions for the regulation of genes (chromatin folding, small RNA, etc.). The readily available group of tools is concerned with modifications of DNA and includes for instance **DNA methylation**, which enables the identification of **biomarkers** to evaluate the condition of individuals and state of populations and species. It is important to remember that DNA methylation is organ‐specific and therefore the accessibility of specific informative tissues matters (Zhou et al., 2017; Zhu et al., 2022). In this review, we will mostly focus on DNA‐based epigenetic methods and their applicability in different areas of conservation (Figure 1). We will build on examples from research on ageing which provides essential information on individuals but also the state of a population (Jackson et al., 2020). We will also focus on species with temperature‐dependent sex determination since their survival is directly linked to the acceleration of climate change (Lockley & Eizaguirre, 2021; Mitchell & Janzen, 2010). Beyond the individual level, determining the signature of environmental effects can act as early warning signals, which can then be integrated into monitoring programmes. Epigenetic markers can also be used to determine the effects of hatcheries/nurseries on species before individuals are released in nature. They can further support ecological restoration and assisted evolution by addressing the preservation and recovery of disrupted ecosystems. While ecological restoration aims to preserve and restore ecosystems in a state before disruption (Jackson & Hobbs, 2009), **(human‐)assisted evolution** describes the improvement of populations by enhancing their stress tolerance – also through genetic modifications (van Oppen et al., 2015).

While many excellent reviews dealing with the descriptions of the different epigenetic mechanisms and their impact on ecological dynamics exist (Goldberg et al., 2007; Husby, 2022; Lamka et al., 2022; Rey et al., 2020), we transfer this knowledge into an applied conservation context. More specifically, we focus on those mechanisms and methods that right now can be harnessed for management and those we anticipate to actively improve future conservation strategies of endangered species and ecosystems. We uphold the recommendation that expanding beyond genomic tools provides a framework to explore novel conservation strategies, such as detecting early signs of selection, monitoring assisted gene flow, and evaluating restoration success.

## 2 INDIVIDUAL‐BASED DNA METHYLATION INFORMATION

**FIGURE 2:**
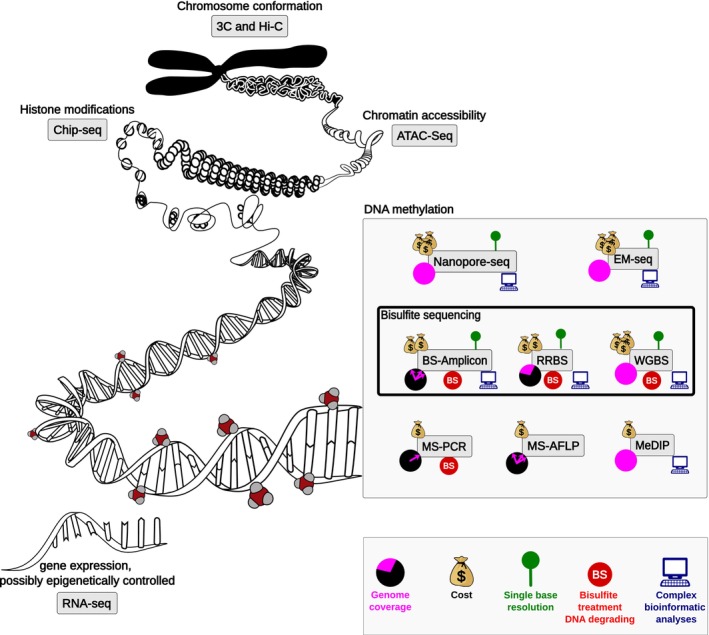
Major tools for detection of epigenetic modifications, with a focus on DNA methylation. Diverse methods provide insights into gene regulation, development, disease, and evolution. Icons represent from left to right of the legend: genome coverage in pink with a black background (one or more genomic positions, part of the genome or full genome); estimated cost (from one bag of money: low costs to three: expensive); specific advantages or disadvantages of the technique represented by the presence of icons for single base resolution, bisulfite treatment which can lead to DNA degradation and complex bioinformatic analyses.

DNA methylation can be assessed using a wide range of methods (Figure 2), including high‐throughput sequencing techniques, such as reduced representation bisulfite sequencing (RRBS), whole‐genome bisulfite sequencing (WGBS) (Chapelle & Silvestre, 2022; Klughammer et al., 2023), or as a by‐product of electric charges of nucleotides using nanopore sequencing (Simpson et al., 2017). To date, the most common approach has been via bisulfite treatment. It converts unmethylated cytosine nucleotides to uracil, while methylated cytosines remain unchanged (Krueger et al., 2012). The bisulfite‐treated DNA is then PCR‐amplified and sequenced to determine the locations of methylated versus unmethylated cytosines (Frommer et al., 1992). By comparing the bisulfite‐treated sequence to the original DNA sequence, the methylation status of cytosine residues can be inferred (Grunau et al., 2001). A disadvantage of bisulfite sequencing (BS‐Seq) is that the bisulfite treatment damages DNA, resulting in fragmentation, loss, and bias (Olova et al., 2018; but see Dai et al., 2024). A promising alternative is an enzyme‐based conversion (e.g. Vaisvila et al., 2021, EM‐Seq), which can be used to detect 5‐mC and 5‐hmC methylation while minimising damage and thus reducing the number of sequencing reads needed. Using examples focusing on ageing and sex determination, we will highlight the potential of those methods for conservation.

### 2.1 Assessing individual age in the wild with epigenetic clocks

Estimating the age of an individual is important as it not only gives an appreciation of this individual's reproductive potential but also its likelihood of living longer in a healthy state. Knowing a population's age structure can further be informative of whether a population is likely to persist in the face of diverse threats (Clutton‐Brock & Sheldon, 2010). In wild animal species, when age determination is possible, this is traditionally done by evaluating annually accrued phenotypic traits, for example, growth rings in fish otoliths (Panella, 1971). Such methods are often lethal, rely on well‐trained experts, may lack precision, and may be impossible to implement in cryptic or endangered species (Campana, 2001). Yet, it is important to monitor wildlife in nature.

While a wide range of molecular age biomarkers have been explored, particularly telomere length, the use of DNA methylation on age‐related genes/genomic regions has progressively become the gold standard (Horvath, 2013; Jarman et al., 2015; Jylhävä et al., 2017). The estimation of chronological age can be made by building models, or “**epigenetic clocks**”, using the DNA methylation values of a subset of **CpG sites** (genomic regions where linear DNA in the 5′ to 3′ direction consists of a cytosine followed by guanine), which strongly correlates with an individual's age (Field et al., 2018; Horvath & Raj, 2018; Parsons et al., 2023).

The DNA methylation‐based ageing technique was originally developed for humans using saliva (Bocklandt et al., 2011), then blood (Hannum et al., 2013), and it was eventually extended to multiple tissues (Horvath, 2013; Levine et al., 2018) – as methylation is cell‐specific and therefore organ‐specific (Lokk et al., 2014). Since then, epigenetic clocks have been developed for a wide range of animals (De Paoli‐Iseppi et al., 2017; Polanowski et al., 2014; see Table S1) and even plants (Gardner et al., 2023; Yao et al., 2023).

Epigenetic clocks can be constructed from a few markers found in age‐correlated genes (i.e., humpback whale *N* = 3 CpGs (Polanowski et al., 2014); long‐lived seabird, *N* = 7 CpGs (De Paoli‐Iseppi et al., 2019); chimpanzee, *N* = 5 CpGs (Ito et al., 2018); Bechstein's bats, *N* = 7 CpGs (Wright et al., 2018)). They can also come from a large set of CpG markers sequenced after cytosine conversion using Illumina sequencing (RRBS, WGBS, EM‐seq; Table 2 and Figure 2), or from custom microarrays such as the widely used custom Infinium array “HorvathMammalMethylChip40” (Arneson et al., 2022), which sequences 36 k loci highly conserved among mammals (pan‐mammalian clock), Lu et al. (2023). In a conservation context, examples include the epigenetic clocks from cetaceans' skin biopsy samples of killer whales (*Orcinus orca*) and bowhead whales (*Balaenus mysticetus*). Retaining >30 k sites, a strong correlation was found between epigenetic and chronological ages, with *R* ^2^ of 0.95 and 0.96 for the killer whale and the bowhead whale, respectively (Parsons et al., 2023). Lastly, the Oxford Nanopore technology (Hayes et al., 2021), which detects changes in electric charge as DNA molecules navigate within nanopores, paves the way for portable in‐field DNA sequencing and methylation, which could be a great asset for conservation biology studies.

**TABLE 2:** List of major tools for detection of epigenetic modification.

Name	Description	Epigenetic mark detected
BS‐seq (Bisulfite sequencing)	DNA is treated with sodium bisulfite, which converts unmethylated cytosines to uracil, while methylated cytosines remain unchanged. After sequencing, the differences in cytosine patterns can reveal the methylation status of specific regions. Within BS‐seq, one can do BSAS (Bisulfite Amplicon Sequencing; Masser et al., 2015), RRBS (Reduced Representation BS; Meissner et al., 2005) and WGBS (Whole Genome BS; Cokus et al., 2008)	DNA methylation
EM‐seq (Enzymatic methyl sequencing)	EM‐seq, developed as an alternative to the damaging BS treatment, uses enzymes, TET2 and T4‐BGT, to protect methylated cytosines, while APOBEC3A converts unmodified cytosines to uracils (Vaisvila et al., 2021)	DNA methylation
Nanopore‐seq (Nanopore sequencing)	DNA passes through nanopores and the resulting electric current is measured. While this method was primarily developed to sequence DNA, modified bases can also be identified due to their specific electric signal (Laszlo et al., 2013)	DNA methylation
MeDIP (Methylated DNA immunoprecipitation)	This method involves using an antibody against methylated DNA to enrich methylated DNA fragments. The enriched DNA is then analysed, often using techniques like microarrays or sequencing (Mohn et al., 2009)	DNA methylation
MSP (Methylation‐specific PCR)	This technique targets specific DNA regions with known CpG islands to determine if they are methylated or unmethylated. It is often used for analysing DNA methylation patterns in specific candidate genes (Ku et al., 2011)	DNA methylation
MS‐AFLP (Methylation sensitive amplified fragment length polymorphism)	DNA methylation on a genome‐wide scale is detected thanks to a combination of methylation‐sensitive restriction enzyme digestion by Not I and the use of amplified fragment length polymorphism (AFLP; Vos et al., 1995; Yamamoto et al., 2001)	DNA methylation
ChIP‐seq (Chromatin immunoprecipitation sequencing)	ChIP‐seq is used to identify DNA regions that are associated with specific proteins, such as histones or transcription factors. It helps to understand the interactions between proteins and DNA, which can influence gene expression (Park, 2009)	Histone modifications
ATAC‐seq (Assay for transposase‐accessible chromatin sequencing)	ATAC‐seq is used to map open chromatin regions, which indicate areas of the genome that are accessible for transcription and regulatory processes. This method can provide insights into gene expression regulation (Buenrostro et al., 2013)	Chromatin accessibility
RNA‐seq (RNA sequencing)	While primarily used to study gene expression at the mRNA level, RNA‐seq can also provide information about epigenetic regulation. Changes in gene expression patterns can be indicative of epigenetic modifications affecting transcription (Nagalakshmi et al., 2008)	Gene expression
3C (Chromosome conformation capture) and Hi‐C (high‐resolution 3C)	These methods analyse the three‐dimensional spatial organisation of chromosomes and help understand how the physical structure of the genome influences gene expression and epigenetic interactions (Dekker et al., 2002; Lieberman‐Aiden et al., 2009)	Chromosome conformation

With such novel methods, we are now in the position to detect correlations between key life‐history traits, such as reproduction and age, which can guide conservation actions, e.g. by protecting reproductive individuals or those about to enter reproduction. Estimates of biological/epigenetic age can also give indications on specific aspects, otherwise overlooked, of the species' evolution. For example, high‐ranking male baboons exhibit accelerated biological/epigenetic ageing compared to their chronological age, indicating a potential trade‐off between high status (correlated with reproductive fitness) and ageing (Anderson et al., 2021). In summary, epigenetic clocks using DNA methylation offer a precise, non‐invasive way to estimate wildlife ages. This knowledge informs conservation efforts and reveals trade‐offs in species' life‐history parameters, guiding conservation strategies.

### 2.2 Assessing individual sex in the case of environmental‐dependent sex determination

Another key individual trait that can be challenging to assess is sex. This is particularly true when there is no sexual dimorphism at birth between sexes and sexing requires complex handling procedures (e.g. invasive laparoscopy) or sacrifice of individuals of endangered species. Over 400 vertebrate species exhibit environmental sex determination (ESD, Lockley & Eizaguirre, 2021), where external factors modify the molecular cascade determining whether an individual becomes a male or a female (Janzen & Phillips, 2006). All sea turtles, crocodilians, and some lizards and fishes present temperature‐dependent sex determination (TSD), while the sex of other fish species can additionally be determined by factors like population density (Capel, 2017; Weber & Capel, 2021) or pH in West African cichlid fish for instance (Reddon & Hurd, 2013).

While sex determination in reptiles has been the focus of intense research, a clear indication of the role of epigenetic mechanisms, more specifically the role of histone H3 lysine 27 demethylase *Kdm6b* (Ge et al., 2018), was revealed in red‐eared slider turtles (*Trachemys scripta elegans*). Knockdown of *Kdm6b* triggers male embryos to revert to females, showing the direct implication of this gene and the resulting enzyme. Another gene involved in TSD in multiple species is the *CYP19* gene coding for the gonadal aromatase, an enzyme crucial for sexual development (Matsumoto et al., 2016; Navarro‐Martín et al., 2011; Parrott et al., 2014). Anastasiadi et al. (2018) used a novel machine‐learning predictive approach based on selected CpG sites to screen aromatase methylation on the European sea bass (*Dicentrarchus labrax*), while Valdivieso et al. (2023) examined the methylation of 15 CpGs in the aromatase gene of zebrafish, which resulted in sex classification with 88% accuracy (Valdivieso et al., 2023). However, it is also possible to use the full genome‐wide DNA methylation information to determine individual sex, instead of pre‐selecting specific genes based on their potential implication in sex determination, as for the American alligators (*Alligator mississippiensis*) (Bock et al., 2022).

From a conservation perspective, as global temperatures increase, models predict the production of extreme sex ratio bias (e.g. Laloë et al., 2014; Lockley & Eizaguirre, 2021), possibly driving species with already small effective population sizes towards extinction. Therefore, being able to determine the sex of individuals at birth can enable species management, for instance, with the use of nurseries to continuously produce the missing sex (Lockley & Eizaguirre, 2021).

### 2.3 Assessing individual traits and responses to environmental conditions

In addition to age and sex, DNA methylation offers insights at the individual level, from life‐history evolution to individual responses to contemporary selective pressures. For example, in the capelin (*Mallotus villosus*), whether an individual is a demersal or beach‐spawner correlates with methylation in the dorsal fin and is partly independent of the underlying genetics (Venney et al., 2023). In killer whales, amplicon bisulfite sequencing from skin samples obtained using a pneumatic dart system showed that anthropogenic stress affects methylation (Crossman et al., 2021). Skin DNA methylation provides an indication of infection by ectoparasites in guppies (*Poecilia reticulata*) (Hu et al., 2018). In wild baboons (*Papio cynocephalus*), food availability affects DNA methylation in blood samples collected under anaesthesia (Lea et al., 2016). Furthermore, DNA methylation in the coral species *Pocillopora damicornis* (Putnam et al., 2016) and *Stylophora pistillata* (Liew et al., 2018) is susceptible to pCO_2_ treatment and could be indicative of ocean acidification effects. Effects of anthropogenic pollution are also traceable with DNA methylation levels, as it has been shown that the blood of great tits (*Parus major*) carries methylation signatures of heavy metal exposure (Mäkinen et al., 2022). Importantly, some environmental stressors can correlate with DNA methylation over several generations as was revealed in zebrafish (*Danio rerio*, Pierron et al., 2021), where cadmium in the mother's gonad influences the sex ratio of offspring.

All these correlations reveal ample opportunities to harness individual‐level DNA methylation patterns to evaluate stress levels in the wild but also of captive individuals in aquaculture, farms, zoos or under‐breeding programs designed to repopulate endangered species. Taken together, these epigenetic markers, ideally identified from tissue samples that can be minimally invasive to collect, allow us to monitor an individual's stress level, or health status and thus, assess a population's tolerance to past and present stressors.

## 3 FROM INDIVIDUALS TO POPULATIONS

Predicting populations' responses to rapidly shifting climatic conditions and to other human‐mediated disturbances is crucial for effective conservation strategies (Eizaguirre & Baltazar‐Soares, 2014). While DNA structural variants have traditionally been the focus of molecular approaches to assess the adaptive potential of species and populations (Eizaguirre & Baltazar‐Soares, 2014; Funk et al., 2019; Hoelzel et al., 2019), recent studies have highlighted the importance of epigenetic variation, particularly in facilitating rapid responses to environmental changes (Lamka et al., 2022; Rey et al., 2020). By analysing epigenetic patterns across populations, it is possible to quantify the relative contribution of epigenetic mechanisms to the adaptive potential in addition to the genetic component. Once this is established, we can identify genetic and epigenetic shifts of key markers in populations, which enables a more accurate approximation of their ability to cope with rapidly occurring stressors (parallel to Hoelzel et al., 2024).

### 3.1 Evolutionary dynamics of the epigenome

At the population level, most studies have focused on DNA methylation and specifically on CpG sites (Heckwolf et al., 2020; Kader & Ghai, 2017; Skvortsova et al., 2018). Those types of DNA marks are relevant because changes in DNA methylation, also known as **epimutations**, can be up to five orders of magnitude more frequent than genetic mutations (10^−4^ versus 10^−9^ per base pair and generation, Schmitz et al., 2011). Therefore, novel epigenetic variation as a means of phenotypic adaptation may provide an accelerated evolutionary pathway (Kronholm & Collins, 2016). This might be particularly important for clonal species, since the higher epimutation rates could compensate for their slower evolutionary rates and lower genetic variation (Lynch et al., 1993) and explain why clonal species thrive in temporally and spatially diverse environments. The example of invasive Japanese Knotweed (*Reynoutria japonica*) has taught us that epigenetic changes can coincide with local microhabitat conditions (Richards et al., 2012), which may allow for locally adaptive phenotypes despite little genetic variation. Further support for this idea comes from a study on a clonal herb (*Hydrocotyle vulgaris*) with low genetic diversity, which found intra‐ and inter‐population epigenetic distance to be the main predictor of phenotypic variation in fitness‐related traits, such as leaf area and petiole length (Wang et al., 2020). While asexually reproducing organisms lack recombination to generate novel genetic diversity, they also do not undergo major epigenetic erasure and can preserve and adjust epigenetic patterns across mitotic reproductive cycles. But also in sexually reproducing species, evidence is accumulating that at least some DNA methylation marks may be stably inherited across generations as suggested in mammals (Li & Zhang, 2014; Zhang & Sirard, 2021), birds (reviewed in Guerrero‐Bosagna et al. (2018), but see Sepers et al. (2023) for the underlying role of genetics), fish (Heckwolf et al., 2020; Wellband et al., 2021) and even insects (Yagound et al., 2020). The facts that DNA methylation (i) responds to environmental stress, (ii) is linked to fitness‐related traits and (iii) can be maintained across generations (Jueterbock et al., 2020) highlight the importance of those markers for eco‐evolutionary dynamics of populations and therefore their relevance for conservation. To evaluate population‐level DNA methylation, two main approaches exist: methylation comparisons across the **epigenome**, and differential methylation comparing specific genomic sites or regions.

### 3.2 Genome‐wide methylation variation as an analogy to genetic diversity

Epigenetic analyses at the population level offer valuable insights into the adaptive potential and extinction risk of species facing rapidly shifting environments. DNA methylation diversity can be measured and, like what would be done with genetic variation, compared among populations. This includes, for instance, counting the total number of genomic sites that are methylated in one population and not methylated in another. Often such dichotomic differences do not exist and instead, thresholds are defined (e.g. higher than 70% or lower than 30%) and counts of sites with methylation values matching these thresholds will be summed up per individual (Sagonas et al., 2020). The thresholds will be chosen based on the model system as different taxonomic groups will have varying overall levels of methylation (Aliaga et al., 2019). Averages among populations can then be estimated and compared. For instance, stickleback infected by the nematode *Camallanus lacustris* showed a higher number of methylated sites than their non‐infected counterparts using RRBS (Sagonas et al., 2020). Another approach consists of directly comparing genome‐wide methylation between populations or groups. In a field experiment investigating the effect of brood size on DNA methylation from RRBS, blood samples of great tits (*Parus major*) showed that nestlings from experimentally enlarged and reduced broods had lower genome‐wide methylation compared to controls (Sepers et al., 2021), matching previous results detected in Zebra finches (*Taeniopygia guttata*, Sheldon et al., 2018).

Similar to quantifying a species' population structure – another component of a species' adaptive potential – it is possible to estimate the epigenetic structures within populations. To understand the role of epigenetics on the population ecology of coastal and offshore ecotypes of bottlenose dolphins, Tatsch et al. (2021) used a methylation‐sensitive amplified polymorphism technique on biopsy samples collected from animals of both ecotypes. They found consistent differences in DNA methylation patterns between coastal and offshore individuals, indicating that the divergence between ecotypes has some epigenetic components. This is important as such studies contribute to the definition of stocks, which may not be otherwise clear from a sole genetic perspective.

Analogous to genetic diversity, genome‐wide methylation variation might play a vital role in predicting populations' adaptability, and ultimately species' survival. DNA methylation tools can guide conservation efforts towards retaining epigenetic variation and delineating conservation units for more effective biodiversity management, which ultimately safeguards the functional integrity of ecosystems.

### 3.3 Differential methylation among wild populations

While informative, global methylation approaches tend to not capture the entire correlations between epigenome and changes in specific traits or with environmental conditions. Hence, methylation ratios are often used to identify sites that are differentially methylated among wild populations. For instance, a link between reproductive traits and DNA methylation variation exists in birds. In the cross‐fostering experiment of great tits, for which brood size had been manipulated, 32 DNA methylation sites were found to be differentially methylated between siblings from enlarged and reduced broods. Those sites were not random regarding their genomic position, since they were located in or near genes involved in development, growth, metabolism, behaviour, and cognition (Sepers et al., 2021). Similar examples are relevant for conservation as those associations identify markers linked to specific traits that can be monitored even if no genetic changes are visible. Here early warning signal of brood size reduction could be captured before the mean brood size of the population decreases. The relationship between epigenomic marks and various fitness‐related traits (e.g. body size, seed production, and parasite load) and even fitness directly has recently been reviewed along abiotic (e.g. temperature) and biotic (e.g. herbivory intensity) gradients (Lamka et al., 2022). The authors argue that epigenetic variation can be inferred to play a role in adaptation if it affects the fitness‐related traits that result from environmental differences. They ultimately propose to utilize these epigenetic marks to predict evolutionary trajectories and inform conservation strategies.

Differential methylation can also capture the effects of environmental change on species. Marine species are particularly affected by heat waves during early development and European sea bass (*Dicentrarchus labrax*) are no exception. Simulated heat waves of up to 3.5C above ambient water temperatures revealed differentially methylated regions (DMRs) around the same genomic regions across tissues of the brain, muscle, liver, and testis, showing metastable **epialleles** (Anastasiadi et al., 2020). Epialleles are defined as the alternative versions of a DNA methylation mark, which can take at least two forms: (i) the presence or absence of a methyl group, e.g. on a cytosine in the CpG context, or (ii) differences in the methylation ratio as a percentage. Those metastable epialleles could constitute a tool to assess global change effects in marine life at a large scale if also conserved across species. Similarly, stickleback fish sampled along a salinity cline showed population‐specific DNA methylation at genes enriched in osmoregulatory processes (Heckwolf et al., 2020). This pattern suggests local adaptation but more importantly here, identifies biomarkers for monitoring the effects of desalinisation of oceans on marine species.

To understand the effect of epigenomic differences on phenotypic traits and thus their relevance for local adaptation, but also the link between epigenomic and environmental variation, we can apply **epigenome**‐wide association studies (EWAS). Similarly to genome‐wide association studies (GWAS), it links epigenomic regions with environmental variables or traits, whether morphologic or physiologic (Hu & Barrett, 2017). The best examples to date come from studies of trees. For example, DNA methylation of valley oak (*Quercus lobata*) showed 43 DNA methylated sites associated with climate variables such as mean maximum temperature. Those sites were associated with genes involved in plant response to the environment (Gugger et al., 2016). By testing the correlation strength between DNA methylation marks or single nucleotide polymorphisms with climate variables, it is even possible to disentangle the respective contribution of epigenetic and genetic variation to adaptive evolution (Gugger et al., 2016; Hu & Barrett, 2017). Overall, EWAS allows us to delineate epigenetic marks underlying environmental adaptation, which is highly relevant to conservation biology.

## 4 ECOLOGICAL RESTORATION AND ASSISTED EVOLUTION OF VULNERABLE ECOSYSTEMS

*Ecological restoration* describes the assisted recovery of degraded or destroyed ecosystems (Jackson & Hobbs, 2009). This process aims to reduce pressures on ecosystems to enable natural regeneration, but also often involves re‐planting or re‐stocking keystone species. In some ecosystems, the mismatch between historical selection pressures and currently changing environmental conditions can make the recovery of an ecosystem to a known historical state impossible (Duarte et al., 2020; Urban, 2015). In such cases, the relocation of species or populations to areas better suited to their ecological needs, so‐called *assisted migration*, can benefit both the organisms and the ecosystems. However, for areas that encounter environmental extremes, selection for tolerance and resistance might have to be conducted in the laboratory – a process known as *(human)‐assisted evolution* (van Oppen et al., 2015). Each of these conservation strategies comes with varying levels of human intervention, implying we must ensure that we do not (i) introduce **maladaptive** alleles into the recipient populations, or (ii) alter their (epi)genetic diversity, but instead aim to (iii) preserve/maximise the adaptive potential of our focal populations. The following paragraphs discuss the relevance of epigenetic tools in achieving these goals.

### 4.1 Epigenetic tools to improve ecological restoration

Re‐planting or re‐stocking is a commonly used method in ecological restoration (Di Sacco et al., 2021; Heffernan & Rushton, 2000; Osathanunkul & Suwannapoom, 2023). Hatchery‐related examples show that rearing conditions affect epigenetic patterns, but also that altered epigenetic marks can persist across life stages and affect germ‐line cells (Koch et al., 2023). Some of the best‐understood cases are linked to the stocking of, for instance, coho salmon (*Oncorhynchus kisutch*), Atlantic salmon (*Salmo salar*), and steelhead trout (*Oncorhynchus mykiss*) (Le Luyer et al., 2017; Nilsson et al., 2021; Wellband et al., 2021). These studies highlight epigenetic modifications induced by captive rearing as a potential explanatory mechanism for reduced fitness in hatchery‐reared salmonids and could explain the high failure rate of reintroduction programmes (McMillan et al., 2023). This effect could stem from maladaptive phenotypes introduced into the populations, despite all efforts to select for adaptive genotypes. Considering the potential heritability of epigenetic marks (Feiner et al., 2022; Heckwolf et al., 2020; Miska & Ferguson‐Smith, 2016; Morgan et al., 1999; Venney et al., 2022; Wellband et al., 2021), these management interventions could have long term negative effects on the recipient populations (Koch et al., 2023). Therefore, we argue that DNA methylation sequencing should be added to the repertoire of management tools for ecological restoration. For example, to optimise hatchery rearing, the validation of least manipulative breeding conditions through epigenome‐wide screening methods (i.e., WGBS, RRBS, EM‐seq; Table 2 and Figure 2) should aim to minimise DNA methylation differences between wild and captive‐bred individuals. In addition, biomarker screens for adaptive epialleles can set the stage for more successful integration of organisms into their local natural habitat. Furthermore, epigenome‐wide screens of captive individuals can also inform about epigenomic diversity, which should be maintained at levels observed in wild populations. In the face of novel environmental conditions, epigenomic diversity could further be increased through diversified bet‐hedging (Angers et al., 2020). This mechanism gives rise to offspring specialised for distinct environments, consequently elevating the probability that some individuals will possess traits suitable for surviving emerging environmental challenges. Ultimately, through epigenetic tools, ecological restoration projects can ensure to maintenance of natural epigenetic patterns and thus long‐term adaptability of populations raised in captive breeding programmes.

### 4.2 Enhancing population resilience through assisted evolution

Restoration projects have often failed, because the pressures that are at the source of increased damage and mortality, such as heat waves, hypoxic events or environmental degradation, are recurrent (Duarte et al., 2020; Herrick et al., 2006). While we argued to aim for the least (epi)genetic divergence introduced by individuals bred in captivity, it might be desirable to strategically introduce adaptive (epi)alleles into threatened populations with low genetic diversity and limited adaptive potential. One method to achieve this is through assisted migration, which involves relocating species or populations to habitats better suited to their ecological needs. This intervention can benefit both the species but also the ecosystem it will be relocated into if the species or population fills an ecological niche that would otherwise be lost (Sansilvestri et al., 2015). Assisted migration has been successfully applied to increase heat tolerance, for instance, by translocating warm‐adapted *Picea glauca* seeds 500 km northwards to colder environments that face increasing temperatures (Sebastian‐Azcona et al., 2019). In the long run, hybridisation between translocated and native organisms should recombine genes considered to be adaptive under future climatic conditions with current locally adapted genes. Similar examples exist, crossing corals from at‐risk reefs with those that come from the hottest reefs in the world. This approach has been shown to increase heat survival by up to 84% (Howells et al., 2021). However, depending on the level of epigenetic and/or genetic divergence between the two parent populations, hybridisation can also lead to (epi)genetic incompatibilities (Laporte et al., 2019), which is one of the most likely problems to occur between populations and species with low levels of divergence. At low prevalence, the resulting fitness disadvantages of hybrids might not be immediately expressed during development. Epigenetic monitoring for aberrant gene silencing or overexpression using RNA or DNA methylation sequencing methods can save valuable time in assessing the potential success of an assisted migration intervention to maintain biodiversity under global warming.

For assisted migration to be applicable, the source ecosystem must be under environmental pressures equivalent to future predictions of the target ecosystem. If such an ecosystem does not exist or has not been described, (human)‐assisted evolution, the selection for tolerance and resistance in the laboratory, could be an alternative (van Oppen et al., 2015). While humans have modified wild animals and plants by domestication (Morrell et al., 2011; Song et al., 2022), the manipulation of wild populations in the context of conservation, i.e. through selective breeding or acclimatisation, is a comparatively novel concept. Only recently, selective breeding has successfully been applied to enhance coral bleaching tolerance by heat‐evolving the microalgal endosymbiont, Symbiodiniaceae (Buerger et al., 2020). The coral itself has also been bred for increased heat tolerance resulting in heritable adaptive genetic variation (Quigley et al., 2020). Similarly, lab acclimatisation and breeding can induce the formation of novel epi‐alleles and select beneficial traits with an epigenetic basis to better withstand environmental stress (Pazzaglia et al., 2021). For example, DNA methylation might play a role in coping with reduced calcification as a response to ocean acidification by reducing transcription noise and fine‐tuning highly expressed genes (Liew et al., 2018). In fish, regardless of the offspring's thermal environment, the temperature experienced during parental sexual maturation explains offspring DNA methylation variation, offering a potential mechanism to improve population resilience to heat waves (Venney et al., 2022). If we can induce epigenetic modifications with beneficial phenotypic effects through controlled stress exposure, we will increase a population's stress tolerance and gain enough time for selection and adaptive responses to occur in the wild.

## 5 FUTURE PERSPECTIVES

### 5.1 To edit or not to edit? State‐of‐the‐art epigenetic modification techniques

The risks of species extinction and the loss of crucial ecosystem functions have prompted studies to explore even invasive management tools. These cutting‐edge epigenome editing techniques use various methods like Zinc finger nucleases (ZNFs), Transcription activator‐like nucleases (TALENs), or modified CRISPR‐Cas9 complexes (Shin et al., 2022; Waryah et al., 2018). Like genetic manipulation tools, these epigenetic modification techniques have made their way into commercial usage. For instance, different modifications of the CRISPR‐Cas9 complex have been used to alter nutritional aspects and increase yield in tomato breeding (Chaudhuri et al., 2022). Interestingly, traits known to be correlated with fitness − such as flowering time, growth rate, and stress tolerance − have also been shown to be changeable through epigenetic modification tools (Jogam et al., 2022; Papikian et al., 2019). Most importantly for the successful application of such tools, the induced methylated state was shown to be meiotically heritable across multiple generations (Papikian et al., 2019). However, targeted epigenetic alterations demand prior knowledge of the specific sequence to be modified. To exploit epigenome editing approaches for conservation, we need a new database or populate existing ones (e.g. OMIA (Nicholas, 2021); Animal QTLdb (Hu et al., 2013); MethHC (Huang et al., 2015)) with information that links epigenetic variations to specific (adaptive) traits. Such resources however only exist for a handful of model organisms, such as *Arabidopsis thaliana*, encompassing extensive **methylomes** from diverse ecotypes, mutants, and epigenetic recombinant inbred lines (Agarwal et al., 2020; Jain et al., 2021). Building such a resource for non‐model organisms will be time and labour‐intensive, if it is possible at all. The complex interplay of epigenomic and genomic information might also affect how transferable these results are from one species to the next. Since species also differ in their ability to inherit epigenetic marks, induced epigenetic modifications will persist in populations with varying success (Carlini et al., 2022; Feiner et al., 2022; Kungulovski et al., 2015). However, even if adaptive epialleles would fade over generations, this could just buy enough time for genetic adaptation to catch up. Besides these shortcomings, epigenetic editing techniques can generally represent an innovative and potentially powerful future tool in the field of conservation biology, as they offer new avenues for preserving endangered species and restoring ecosystems on the verge of collapse. However, it is essential to carefully consider the ethical implications and potential risks associated with these techniques, which must be discussed with a wide audience of experts (Filbee‐Dexter & Smajdor, 2019; Ricciardi & Simberloff, 2009).

### 5.2 Beyond DNA methylation − the future of epigenetic markers?

Throughout this review, we have mostly referred to DNA methylation as the 5‐methylcytosine (**5mC**), which is the most studied modification, but not the only one. There are other types of DNA methylation, such as 5‐hydroxymethylcytosine (5hmC), an intermediate step to the demethylation of 5mC. This epigenetic marker is stable and used as a biomarker for human cancer (Li et al., 2017). It could further be useful for age determination as it accumulates with age in mammals (Chouliaras et al., 2012; Hahn et al., 2013).

Other epigenetic markers like histone modifications and non‐coding RNAs (**ncRNAs**) have also been characterised but are currently unused resources for conservation efforts. As of today, histone modification and ncRNAs have proven their value as diagnostic biomarkers in cancer research, with significant therapeutic potential (Jung et al., 2020). Furthermore, the abundance of ncRNAs also changes with age (Kim et al., 2021; Wang et al., 2022). However, since DNA methylation‐based age biomarkers offer much higher prediction accuracy (Zbieć‐Piekarska et al., 2015), they remain the gold standard. Similarly, ubiquitylation of long‐lived histone 2A proteins shows an evolutionarily conserved, age‐related increase in species such as *Drosophila*, mice, monkeys, and humans, making it an interesting interspecific age biomarker candidate (Yang et al., 2019). Beyond age and cancer, histone modifications are also sensitive to diet and can be informative of an individual's health status (Molina‐Serrano et al., 2019; Upadhyaya et al., 2017). In the field of assisted evolution, conceptual papers mention histone modifications and ncRNAs when defining the set of existing epigenetic marks (van Oppen et al., 2015). However, thus far, examples demonstrating potential applicability as tools in restoration ecology or assisted evolution are still missing. We advocate that this avenue should be explored with a specific focus on evaluating the potential practicality of histone modification and ncRNA‐based tools for conservation biology, particularly given the current limitation in sample preservation and processing (i.e. samples for histone modification studies need to contain large numbers of cells and be handle fresh or flash‐frozen; RNA degrades quickly; Ladd‐Acosta, 2015).

### 5.3 Integrating epigenetics with ecological modelling

To fully exploit the potential of population‐level epigenetic analyses, integration with ecological models is essential. Incorporating epigenetic data into existing species distribution models or population viability analyses (PVA) can enhance predictions of species' responses to future climate scenarios. Species distribution models can be extended to incorporate epigenetic data as additional environmental predictors, improving the accuracy of species distribution projections. By considering the effects of epigenetic factors on demographic processes and adaptation, PVA provide more accurate estimations of extinction risk. PVA can incorporate epigenetic effects on fitness and examples found that including epigenetic factors improved predictions of population persistence. In this context, Baltazar‐Soares et al. (2023) proposed a framework to integrate genomic information on temporal projections of biodiversity distribution computed by Species Distribution Models. If dynamic epigenetic marks are used instead of genetic markers, then one can simultaneously conduct monitoring and run models at regular intervals to dynamically manage fish stocks. This approaches the digital twin philosophy where dynamic epigenetic marks can reveal quick spatial and/or temporal changes due to changing selection pressures.

### 5.4 Epi‐environmental DNA

Environmental DNA (**eDNA**) is a monitoring tool that relies on capturing and sequencing DNA molecules present in environmental samples, which can be soil, sediment or the air (e.g. Thomsen & Willerslev, 2015). Due to its non‐invasive nature, sampling does not require the extraction of biological tissue from living organisms. It is the tool of choice to (i) investigate the occurrence of endangered taxa or (ii) obtain presence/absence information on cryptic or elusive species (for example, predators) and (iii) characterise entire communities. The potential of eDNA can however be expanded.

eDNA monitoring strategies have a limited scope to investigate the adaptive potential and short‐term biological responses to environmental disturbance. Since epigenetic information identifies quick changes in organismal regulatory pathways – as expected under stress conditions – expanding established eDNA assays to **epi‐eDNA** is an expected upgrade to molecular‐based monitoring activities. Screening for markers associated with gene regulation is not necessarily novel: Cristescu (2019) proposed RNA as a regulatory environmental biomarker. The major setback of the molecule however is its short‐lived nature in the environment (Yates et al., 2021). Contrary to RNA transcripts, DNA methylation marks are stable and as such, could still be detected when shed from the organism (Sigsgaard et al., 2020).

The technological leap necessary to cover the gap between traditional eDNA analysis and epi‐eDNA analysis has recently advanced. However, it has been largely applied to human medical research. Perhaps the most known strategy is methylation‐sensitive qPCR (MS‐qPCR) used in the detection of 5mC patterns of the **promoter regions** of key tumour‐related genes from liquid biopsies, blood samples, or formalin‐preserved tissue (Beikircher et al., 2018; Bendixen et al., 2023; De Chiara et al., 2020; Munson et al., 2007; Wiencke et al., 2014). To the best of our knowledge, it has not yet been published for screening environmental samples. Similar to MS‐qPCR, droplet digital PCR (ddPCR) has been routinely utilised in medical research. However, Zhao et al. (2023) showed its potential to measure methylation in an eDNA context focusing on four different life stages of the great pond snail (*Lymnaea stagnalis*) as examples. DNA shed from the organism and tissue (skin) were screened with RRBS for methylation marks, which enabled cross‐validation. Results showed that epi‐eDNA characterised life stages (as methylated DNA patterns change with age). If environmental samples can retain information related to species, monitoring expands from identifying cryptic species to gaining individual information.

The decision‐making process before engaging in epi‐eDNA analyses will likely be dominated by the following questions: (a) Do we want to monitor signs of individual and population health in addition to diversity? (b) Shall we focus on specific genes or the overall methylome? The challenge is linked to targeting conserved regions with adaptive value, for instance, epialleles being sensitive to environmental change. The most plausible targets are promoter regions of genes known to be sensitive to the stressor. For example, environmental pollution triggers a series of DNA methylation changes both at global and gene‐specific scales in invertebrates (Šrut, 2021). These could be suitable starting points to develop species or taxonomic group‐specific epi‐eDNA tools for common stressors. On the other hand, monitoring entire communities in an epi‐metabarcoding effort would require regulatory non‐coding regions to be taxonomically conserved. There exist databases, such as UCNEbase, Ancora, dbCNS, and VISTA, compiling knowledge on ultra‐conserved non‐coding elements from various species that can form a starting point to mine for putative epi‐eDNA target molecules (Dimitrieva & Bucher, 2013; Engström et al., 2008; Inoue & Saitou, 2021; Visel et al., 2007).

Though promising, epi‐eDNA comes with limitations, similar to eDNA: false positives and false negatives. How to deal with these specific issues in eDNA research has been elaborated upon (see Sigsgaard et al., 2020; Skelton et al., 2022; Yao et al., 2022 to name a few). Yet, for epi‐eDNA, traditional challenges might be multifold. This is because epigenetic marks in general (methylation included) are not only tissue‐, age‐, and condition‐specific, but also species‐ and even population‐specific. Therefore, it might not be possible to track from which organ DNA originated and what signals are detected, or they might not be detected across conspecifics, if they are from different life stages, age cohorts, etc. There is also the issue of deamination or the degradation of methylated cytosines into thymine due to the harsh extracellular environment − expectedly biassing rates of false‐negatives. Proposed risk mitigation strategies range from having good reference genomes, and assisting in true‐base calling, to the construction of methylation maps (for various species and tissues, Blake et al., 2020; Schadewell & Adams, 2021; Sigsgaard et al., 2020).

## 6 CONCLUSIONS

Exploring beyond the traditional genetic landscape for novel ways to understand organismal adaptation is critical in the current context of biodiversity loss and fast‐paced shifting climatic conditions. Here, we have outlined how technological advances in the identification of epigenetic marks allied with developed know‐how on physiology and functionality of molecular pathways beyond traditional model organisms have the potential to be efficiently implemented in biodiversity monitoring strategies. Though we discussed readily available epigenetic tools, which can be integrated into conservation projects, we acknowledge that many research avenues remain to be explored. For example, the major focus on 5mC DNA methylation simply implies that more empirical evidence is needed to know whether other types of epigenetic marks can become viable markers. We argue that only when the functionality of epigenetic marks is well‐understood, it becomes appropriate (and safe) to apply them. Lastly, we peeked into the future to spike interest among conservation‐driven researchers interested in paving the way for the growing field of conservation epigenetics.

## FUNDING INFORMATION

A.B is funded by European Union's Horizon 2020 research and innovation programme under the Marie Skłodowska‐Curie grant agreement No. 101026703. M.B.S. is funded by the Academy of Finland (Funding decision 321471). C.E is supported by UK Research and Innovation (NERC, NE/V001469/1, NE/X012077/1). MJH is funded by the German Research Foundation through their Walter Benjamin programme (HE 8763/1‐1 and 8763/2‐1).

## CONFLICT OF INTEREST STATEMENT

Alice Balard, Miguel Baltazar‐Soares, and Melanie Heckwolf are Editorial Board members of Evolutionary Applications and co‐authors of this article. To minimize bias, they were excluded from all editorial decision‐making related to the acceptance of this article for publication.

## Supporting information


Table S1

## Data Availability

The data that supports the findings of this study are available in the supplementary material of this article.
